# Aberrant insulin receptor expression is associated with insulin resistance and skeletal muscle atrophy in myotonic dystrophies

**DOI:** 10.1371/journal.pone.0214254

**Published:** 2019-03-22

**Authors:** Laura Valentina Renna, Francesca Bosè, Elisa Brigonzi, Barbara Fossati, Giovanni Meola, Rosanna Cardani

**Affiliations:** 1 Laboratory of Muscle Histopathology and Molecular Biology, IRCCS-Policlinico San Donato, San Donato Milanese, Milan, Italy; 2 Department of Biomedical Sciences for Health, University of Milan, Milan, Italy; 3 Department of Neurology, IRCCS-Policlinico San Donato, San Donato Milanese, Milan, Italy; Tohoku University, JAPAN

## Abstract

Myotonic dystrophy type 1 (DM1) and type 2 (DM2) are autosomal dominant multisystemic disorders linked to two different genetic loci and characterized by several features including myotonia, muscle atrophy and insulin resistance. The aberrant alternative splicing of insulin receptor (IR) gene and post-receptor signalling abnormalities have been associated with insulin resistance, however the precise molecular defects that cause metabolic dysfunctions are still unknown. Thus, the aims of this study were to investigate in DM skeletal muscle biopsies if beyond *INSR* missplicing, altered IR protein expression could play a role in insulin resistance and to verify if the lack of insulin pathway activation could contribute to skeletal muscle wasting. Our analysis showed that DM skeletal muscle exhibits a lower expression of the insulin receptor in type 1 fibers which can contribute to the defective activation of the insulin pathway. Moreover, the aberrant insulin signalling activation leads to a lower activation of mTOR and to an increase in MuRF1 and Atrogin-1/MAFbx expression, possible explaining DM skeletal muscle fiber atrophy. Taken together our data indicate that the defective insulin signalling activation can contribute to skeletal muscle features in DM patients and are probably linked to an aberrant specific-fiber type expression of the insulin receptor.

## Introduction

Myotonic dystrophy type 1 (DM1) and type 2 (DM2) are autosomal dominant multisystemic disorders caused by expansion of microsatellite repeats. DM multisystemic involvement includes myotonia, progressive muscle wasting, cardiac conduction defects, cataracts, neuropsychiatric disturbances and metabolic dysfunctions. DM1 is caused by an expanded (CTG)n in the 3’ untranslated region of *Dystrophia Myotonica Protein Kinase* (*DMPK*) gene, while DM2 is caused by an expanded (CCTG)n in the intron 1 of *CCHC-type zinc finger*, *nucleic acid-binding protein (CNBP)* gene [1–5]. In both forms, the mutant transcripts accumulate as nuclear foci altering the function of several splicing regulators, leading to aberrant alternative splicing of different genes that explain different DM phenotypic features [6–12]. Insulin resistance is the principal metabolic abnormality of DM patients, which are also characterized by other metabolic dysfunctions such as hyperinsulinemia, hypertriglyceridemia and increased fat mass. Insulin resistance is an important factor for the development of Type 2 Diabetes Mellitus (T2DM) and a risk factor for atherosclerosis, hypertension, cardiovascular diseases, neuropathy, obesity and loss of muscle mass. Thus, insulin resistance represents a pathological condition that can contribute to worsen some aspects of the multisystemic phenotype of DM patients. To date insulin resistance was associated to *INSR* misplicing [10], however, more recently post-receptor defects in insulin signalling [13] has been described. It is known that insulin plays an important role in skeletal muscle growth, development, differentiation and regeneration and that in absence of insulin stimulus, a loss in muscle mass and strength can be observed [14–15]. Progressive muscle wasting and weakness are important characteristic features of both DM1 and DM2, but to date no mechanistic explanation for skeletal muscle histopathological alterations has been described. Unlike other forms of muscular dystrophy, fiber atrophy is not accompanied by fiber regeneration and degeneration in DM and muscle fibrosis are present only in the end stage disease [16]. Previous studies have shown that muscle atrophy in DM patients could reflect an altered balance between muscle protein synthesis and degradation suggesting that muscle wasting could be a result of a defect in muscle metabolism [16–18]. Thus, the aims of this study were to further investigate in DM *ex vivo* muscle biopsies if beyond *INSR* missplicing, altered insulin receptor (IR) expression could play a role in insulin resistance and to verify if the lack of insulin pathway activation could contribute to skeletal muscle atrophy. To specifically address these issues, insulin action on its signalling and on those pathways that regulate protein metabolism was analysed on isolated skeletal muscle fragments obtained from a muscle biopsy according to a well established method previously used to study insulin resistance and T2DM in human and mice model [19–21]. One advantage of this approach is that insulin action, expression of insulin pathway components and muscle dystrophic changes can be studied on the same muscle sample, thus clarifying the exact contribution of insulin signalling in DM skeletal muscle molecular and morphological alterations. The results of this study add more mechanistic insights into insulin resistance condition and skeletal muscle wasting in DM patients, thus representing an improvement in designing effective treatment.

## Materials and methods

The study protocol was reviewed and approved by the ethical committee Ospedale San Raffaele (Milan, Italy) and was conducted according to the principles expressed in the Declaration of Helsinki, the institutional regulation and Italian laws and guidelines. Written informed consents were obtained from the patients for all blood samples and muscle biopsies used in this study.

### Patients and skeletal muscle samples

Skeletal muscle biopsies were taken under sterile conditions from a total of 8 DM1 (7 *tibialis anterior* and 1 *biceps brachii*) and 3 DM2 patients (3 *biceps brachii*) enrolled in The Italian Registry for Myotonic Dystrophy Type 1 and Type 2. In DM2 patients, muscle biopsies were performed for diagnostic purposes while in DM1, muscle biopsies were obtained for research intents. Three age-matched subjects with no sign of neuromuscular disease were used as healthy controls (CTR) (3 *biceps brachii*). As internal controls, three subjects affected by motor neuron disease (MND) (1 *biceps brachii* and 2 *vastus lateralis*) and three subjects affected by Type 2 Diabetes Mellitus (T2DM) (2 *vastus lateralis* and 1 *biceps brachii*) were used. The diagnosis of DM was based upon the clinical diagnostic criteria set by the International Consortium for Myotonic Dystrophy [22]. DM1 and DM2 genotyping was performed on genomic DNA extracted from peripheral blood leukocytes as previously described [23–24]. DM2 diagnosis was performed by fluorescence in situ hybridization on muscle frozen sections using a (CAGG)_5_ probe as previously reported by Cardani et al. [25] to verify the presence of nuclear accumulation of mutant RNA. All patients underwent overnight fasting before blood and muscle samples collection. Skeletal muscle biopsies were fresh-frozen in isopentane cooled in liquid nitrogen. Histopathological analysis was performed on serial sections (8 μm) processed for routine histological or histochemical staining [26]. Small fresh muscle fragments were used for insulin pathway analysis (see below).

### Immunohistochemistry and morphometric analysis

Immunohistochemical staining was performed on serial sections (6 μm) air-dried and rehydrated in phosphate buffer solution pH 7.4 (PBS). Non-specific binding sites were blocked with normal goat serum (NGS; Dako, Glostrup, Denmark) at a dilution 1:20 in PBS containing 2% bovine serum albumin (BSA; Sigma-Aldrich, St. Louis, MO, USA) for 20 min at room temperature (RT). Sections were then incubated for 1 h with mouse monoclonal primary antibodies against two different myosin heavy chain (MHC): MHCslow (1:400 in PBS+2%BSA, Sigma-Aldrich) which recognises type 1 fibers and MHCfast (1:400 in PBS+2%BSA, Sigma-Aldrich) which recognises type 2. Sections were washed in PBS for three times (5 min each) and then incubated for 1 h with goat anti-mouse biotinylated secondary antibody (1:300 in PBS+2%BSA). After washing in PBS (3x5 min), sections were incubated for 30 min with Vectastain ABC complex (Vector Laboratories, Burlingame, CA, USA) and then with 3,3’-Diaminobenzidine (DAB) and hydrogen peroxide for 20 min. Finally, nuclei were counterstained with Mayer’s hematoxylin. Quantitative evaluation of fiber diameter was made as described previously by Vihola et al. [27] on images taken with a Slide Scanner NanoZoomer-S60 (Hamamatsu Photonics, Japan) using the slide scanner software NanoZoomer Digital Pathology NDP. The size of muscle fibers was assessed by measuring the ‘‘smallest fiber diameter.” All data were elaborated using Microcal Origin (Microcal Software Inc., Northampton, MA, USA). On the same images, the quantitative evaluation of fast and slow fiber area was made using ImageJ (Scion Co.). The percentage of fast or slow area on total muscle area was calculated.

### Immunofluorescence

Immunofluorescence staining was performed on serial sections (6 μm) air-dried and rehydrated in phosphate buffer solution pH 7.4 (PBS). Non-specific binding sites were blocked with NGS (Dako-Cytomation) at a dilution 1:20 in PBS+2% BSA for 20 min at room temperature. Sections were then incubated overnight at 4°C with a mouse monoclonal anti-insulin receptor β primary antibody, which recognises both IR-A and IR-B isoforms (18–44, Abcam, 1:100 in PBS+2%BSA). After washing in PBS (3x5 min), sections were incubated for 1 h at room temperature with the secondary antibody (goat anti-mouse Alexa 488-labeled; Molecular Probes, Life Technologies, Milan, Italy; 1:400 in PBS+2%BSA). After washing in PBS (3x5 min), nuclei were stained with DAPI (Sigma-Aldrich). Skeletal muscle sections were finally mounted with Mowiol and examined using a fluorescence microscope (Zeiss Axio Imager M1).

### Alternative splicing of insulin receptor

Skeletal muscle biopsies were mechanically lysed in 1 ml of TRIzol reagent (Gibco BRL, Gaithersburg, MD) using Tissue Lyser (Qiagen). 1 μg of total RNA was reverse transcribed to cDNA using SuperScript III First-Strand Synthesis System (Invitrogen, Thermo Scientific, Rockford, USA) following manufacturer’s instruction. The *INSR* gene was amplified by classical RT-PCR using gene specific primers: forward primer 5’-CCAAAGACAGACTCTCAGAT-3’ and reverse primer 5’-AACATCGCCAAGGGACCTGC-3’. Each PCR reaction was performed in triplicate using Platinum Taq (Thermo Scientific, Rockford, USA) according to manufacturer’s protocol. Total RT-PCR products were electrophoretically resolved on 2,5% agarose gel. Qualitative analysis of the alternative splicing of *INSR* was performed using EtBr-stained gel (Sigma-Aldrich, St. Louis, MO) scanned on a ChemiDoc Universal Hood (BioRad). Quantitative analysis was performed quantifying the intensity of each band with ImageJ software densitometry and calculating the proportions of normally spliced (*INSR-B*) isoform respect to the total amount of the isoforms.

### Insulin stimulation of isolated skeletal muscle and protein extraction

The method to study the response to insulin action in *ex vivo* skeletal muscle biopsies obtained from DM1 and DM2 patients was similar to that described previously on human T2DM muscle with some modifications [19–21].

Briefly, small fresh muscle fragments (10 mg) have been pre-incubated for 30 minutes at 37°C in 2 mL of a daily prepared modified Krebs–Henseleit buffer (KHB) and then they have been incubated for 20 minutes at 37°C in 2 mL KHB supplemented or not with 10nM insulin and rapidly cooled in liquid nitrogen. Muscle samples have been then processed for whole cell protein extraction. Muscle fragments homogenized in 100 μl of 50 mM TrisHCl with 5% SDS (pH 7.5) supplemented with protease and phosphatase inhibitors using Tissue Lyser (Qiagen). After incubating on ice for 15 min, samples were centrifuged at 5700 g for 15 min at 4°C and supernatant was collected and stored at -80°C. Protein concentration was determined with Pierce BCA Assay Kit (Thermo Scientific, Rockford, USA).

### Western blot analysis

40 μg of total proteins were separated by SDS-PAGE and transferred to nitrocellulose membranes. After blocking non-specific binding with TrisHCl buffer pH 7.5 (TBS) containing 5% BSA, the membranes were incubated with primary antibodies diluted in TBS+5%BSA+0,3%Tween20. After washing with TBS+0.3% Tween20, membranes were incubated with HRP-conjugated anti-mouse or anti-rabbit secondary antibodies (Jackson ImmunoResearch Laboratories, INC) diluted 1:5000 or 1:10000 in TBS+5%BSA+0,2%Tween20 respectively. Super signal West Pico Chemiluminescent Substrate (Thermo Scientific, Meridian Rd., Rockford, USA) was used for immunodetection. For western blot application, the following primary antibodies were used: mouse monoclonal anti-Insulin Receptor β (18–44), which recognises both IR-A and IR-B isoforms, (Abcam, 1:500); rabbit polyclonal anti-IRS1 phospho Tyr612 (Abcam, 1:300); rabbit polyclonal anti-IRS1 (Abcam; 1:500); mouse monoclonal anti-Akt phospho Thr308 (clone L32A4, Cell Signaling Technology; 1:500); rabbit monoclonal anti-Akt phospho Ser473 (clone 193H12, Cell Signaling Technology; 1:1000); rabbit polyclonal anti-Akt (Cell Signaling Technology; 1:2000); rabbit polyclonal anti-p70S6 kinase phospho Thr421/Ser424 (Cell Signaling Technology; 1:1000); rabbit polyclonal anti-p70S6 kinase (Cell Signaling Technology; 1:500); rabbit polyclonal anti-p44/42 MAPK (Erk1/2) phospho Thr202/Tyr204 (Cell Signaling Technology; 1:1000); mouse monoclonal anti-p44/42 MAPK (Erk1/2) (clone 3A7, Cell Signaling Technology; 1:2000); rabbit monoclonal anti-AS160 phospho Thr642 (clone D27E6, Cell Signaling Technology; 1:1000); rabbit monoclonal anti-AS160 (clone C69A7, Cell Signaling Technology; 1:1000); rabbit polyclonal anti-FoxO1 phospho Thr24 (Cell Signaling Technology; 1:1000); mouse monoclonal anti-FoxO1 (clone D7C1H, Cell Signaling Technology; 1:1000); rabbit polyclonal anti-mTOR phosphor Ser2448 (Cell Signaling Technology; 1:500); rabbit polyclonal anti-mTOR (Cell Signaling Technology; 1:500); rabbit monoclonal anti-Fbx32 (clone EPR9148(2), Abcam; 1:2000); rabbit polyclonal anti-MuRF1 (Abcam; 1:2000). All the phosphorylated isoforms analysed in this study correspond to an active form of the protein, except for FoxO1 phosphorylation which correspond to inactive form of the protein. Protein activation or inactivation was evaluated as phosphorylation/total ratio. Rabbit polyclonal anti-GAPDH (Cell Signaling Technology; 1:10000) was used as an internal loading control. Each experiment was performed in triplicate and quantitative analysis was performed quantifying the intensity of each band with ImageJ software densitometry.

### Statistical analysis

Statistical analysis was performed using GraphPad Prism software, version 7 (GraphPad Software Inc., CA, USA). For comparison between two groups Student’s *t*-test was used. To measure the association between variables a Pearson’s Correlation Coefficient was used. The differences were considered statistically significant at p<0.05.

## Results

### Patients

This study was performed on skeletal muscle biopsies obtained from 8 DM1, 3 DM2 patients and 3 subjects with no sign of neuromuscular disease used as controls (CTR). As internal controls, 3 patients affected by motor neuron disease (MND) as a neurogenic model of skeletal muscle atrophy and 3 patients affected by Type 2 Diabetes Mellitus (T2DM) as a model of insulin resistance were used (Table 1). The DM1 cohort was represented by classical adult patients mildly affected with a range of CTG repeat expansion of 396±180, while the DM2 cohort was represented by 3 patients with classical proximal myotonic myopathy (PROMM) phenotype. Among T2DM patients, T2DM-2 was treated with metformin. To allow the comparison between data obtained on skeletal muscle biopsies, all samples were taken in the morning after overnight fasting.

**Table 1:** Clinical data on CTR, MND, T2DM and DM patients used in this study.

Patient	Sex	Muscle	Age at biopsy	CTG repeat size	MRC^a^	MIRS [28]^b^	HOMA (<2,5)^c^	BMI^d^	%EF^e^	PR^f^	QRS^f^
**CTR-1**	F	BB	58	/	127	/	1.7	22.7	n.d.	n.d.	n.d.
**CTR-2**	M	BB	34	/	130	/	1.5	25.0	n.d.	n.d.	n.d.
**CTR-3**	F	BB	32	/	128	/	n.d.	23.4	n.d.	n.d.	n.d.
**MND-1**	F	BB	65	/	120	/	n.d.	17.6	n.d.	n.d.	n.d.
**MND-2**	M	VL	43	/	114	/	2.3	24.2	n.d.	n.d.	n.d.
**MND-3**	M	VL	64	/	130	/	1.8	22.6	67	184	102
**T2DM-1**	F	VL	34	/	130	/	6.3	26.1	n.d.	n.d.	n.d.
**T2DM-2**	F	VL	63	/	130	/	9.8	27.2	n.d.	n.d.	n.d.
**T2DM-3**	M	BB	50	/	130	/	7.1	26.6	n.d.	n.d.	n.d.
**DM1-1**	F	TA	39	300	125	4	1.3	29.93	54	192	130
**DM1-2**	F	TA	34	250	126	3	1.2	20.44	64	174	86
**DM1-3**	F	TA	24	300	129	2	2.4	20.2	52	233	102
**DM1-4**	F	TA	36	630	112	4	1.6	20.58	58	158	92
**DM1-5**	F	TA	34	550	124	3	1.4	21.2	56	186	94
**DM1-6**	M	BB	56	230	120	3	5.3	26.1	54	208	102
**DM1-7**	M	TA	24	800	129	5	2.3	28.3	50	176	102
**DM1-8**	F	TA	22	250	126	2	2.5	19.71	68	166	90
**DM2-1**	F	BB	61	/	122	/	1.9	24.6	69	160	72
**DM2-2**	F	BB	36	/	128	/	1.3	21.4	70	164	79
**DM2-3**	M	BB	49	/	125	/	2.4	24.4	87	156	87

CTR, control; MND, motor neuron disease; T2DM, type 2 diabetes mellitus, DM1, myotonic dystrophy type 1; DM2, myotonic dystrophy type 2; F, female; M, male; BB, biceps brachii; VL, vastus lateralis; TA, tibialis anterior.

^a^Medical Research Council, scale for muscle strength; scale (0–5 grade) on 13 muscles at both sides in the upper and lower limbs for a total of 130 maximum score.

^b^Muscle Impairment Rating Scale, stage of the disease in myotonic dystrophy type 1 (DM1) patients.

^c^HOMA (HOmeostasis Model Assessment) was calculated using the formula: HOMA = [glucose (mg/dl) x insulin (μU/Ml)/405], using fasting values.

^d^Body Mass Index.

^e^Cardiac Ejection Fraction, normal values >50%.

^f^Electrocardiographic abnormalities: normal PR interval <200ms, normal QRS duration <100ms.

### Skeletal muscle histopathology

**Fig 1:**
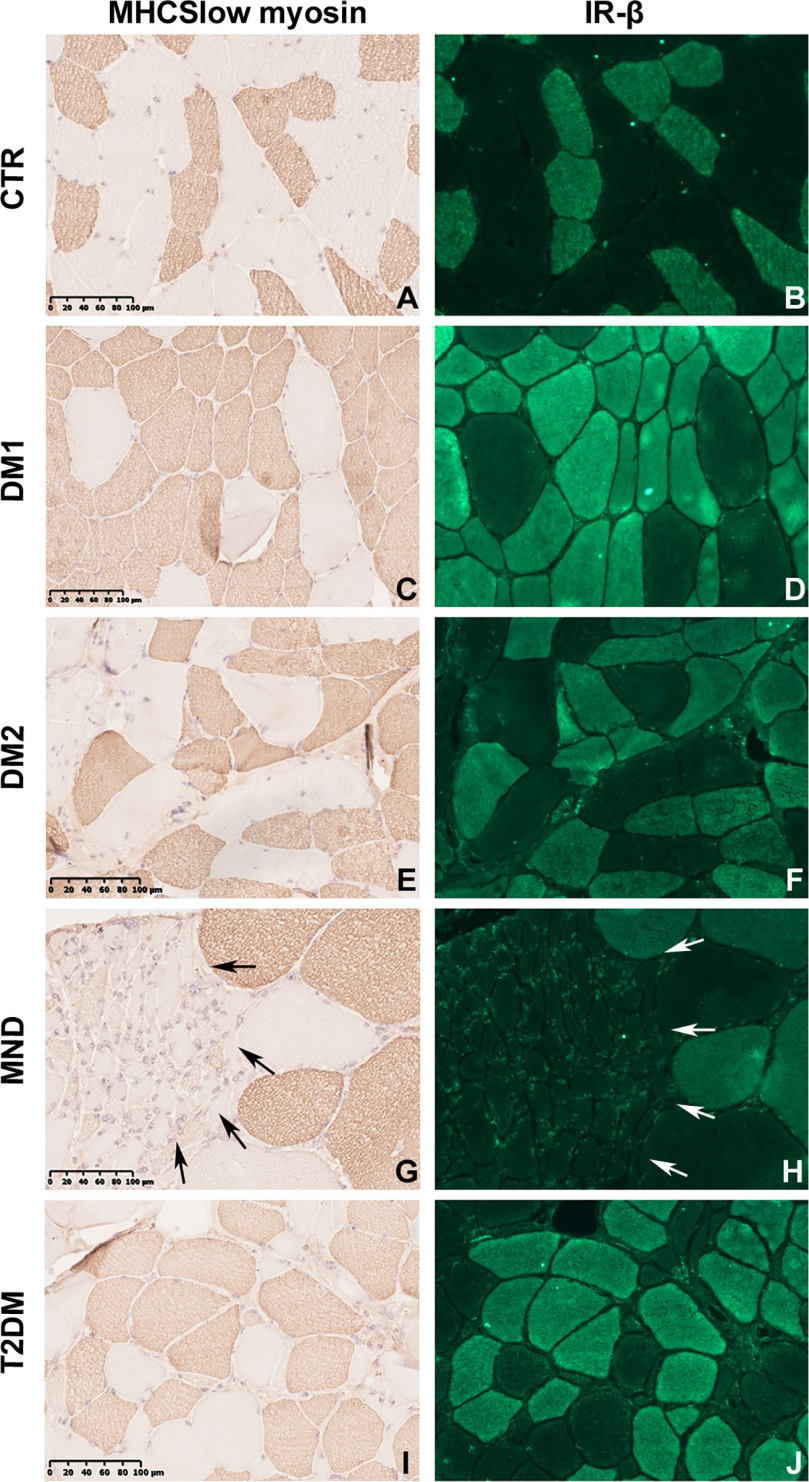
MHCslow immunostaining and insulin receptor immunolocalization. Representative microscopy images from serial skeletal muscle sections stained with antibodies against MHCslow (brown) or IR (green). Images are representative of 1 CTR **(A, B)**, 1 DM1 **(C, D)**, 1 DM2 **(E, F)**, 1 MND **(G, H)** and 1 T2DM **(I, J)** patient. In (g) and (h) arrows define type 2 fiber grouping. Original magnification 200x.

Routine stainings performed on skeletal muscle transverse sections revealed the presence of the most common histological alterations observable in DM skeletal muscles such as nuclear clump fibers, nuclear centralization and fiber size variability with the presence of atrophic and hypertrophic fibers. Stainings performed on MND patients showed the classic features of motor neuron diseases such as atrophy and compensatory hypertrophy of both fiber types and areas with fiber type grouping. As expected, also T2DM patients showed fiber size variability due to fiber atrophy, while no alterations were found in CTR skeletal muscles. The analysis of muscle sections immunostained for MHCfast or MHCslow myosin allowed us to detect and measure fibers smaller than 5 μm, including all nuclear clump fibers which are recognizable by the presence of a thin rim of immunoreaction around the nuclei, and thus to better evaluate type 1 and type 2 fiber atrophy and hypertrophy (Fig 1A, 1C, 1E, 1G and 1I). An increase of type 1 and/or type 2 fiber atrophy was present in almost all DM1 patients (Fig 1C, Table 2), while a selective type 2 fibre atrophy was evident in DM2 patients (Fig 1E, Table 2). Hypertrophy of type 1 and type 2 fibers were also present in DM1 patients (Fig 1C, Table 2). Morphometrical analysis of MND immunostained sections revealed a marked fiber atrophy involving preferentially type 2 fibers (Fig 1G, Table 2). In T2DM patients fiber atrophy was present in both type 1 and type 2 fibers (Fig 1I, Table 2).

**Table 2:** Atrophy and hypertrophy factors of skeletal muscles used in this study.

	AF	AF-FAST	AF-SLOW	HF	HF-FAST	HF-SLOW	% type 1 fiber area
**CTR-1**	0.21	0.18	0.23	0.22	0.28	0.15	28.6%
**CTR-2**	0.13	0.09	0.16	1.69	1.75	1.63	9.2%
**CTR-3**	0.16	0.15	0.17	0.26	0.32	0.21	24.0%
**MND-1**	5.02	8.24	1.79	0.22	0.28	0.15	39.3%
**MND-2**	7.28	14.56	0.00	2.50	0.33	4.67	35.9%
**MND-3**	4.62	5.79	3.45	0.00	0.00	0.00	45.0%
**T2DM-1**	1.02	0.53	1.50	0.00	0.00	0.00	26.0%
**T2DM-2**	3.47	6.52	0.41	0.52	0.21	0.83	36.5%
**T2DM-3**	0.26	0.19	0.34	0.19	0.25	0.13	20.0%
**DM1-1**	0.41	0.51	0.30	14.66	22.39	6.92	84.1%
**DM1-2**	1.72	0.33	3.10	1.93	3.70	0.16	62.3%
**DM1-3**	1.12	0.23	2.01	3.04	5.53	0.55	36.9%
**DM1-4**	1.58	1.90	1.25	4.20	5.52	2.88	91.9%
**DM1-5**	1.30	2.46	0.13	6.55	7.02	6.07	81.8%
**DM1-6**	0.28	0.16	0.39	0.66	1.00	0.32	33.3%
**DM1-7**	2.07	3.92	0.22	1.96	1.53	2.38	91.5%
**DM1-8**	0.64	0.00	1.28	6.17	11.40	0.94	72.4%
**DM2-1**	1.57	2.45	0.69	0.74	1.10	0.37	61.3%
**DM2-2**	2.60	4.59	0.60	0.02	0.01	0.03	49.9%
**DM2-3**	3.67	7.14	0.19	2.13	1.58	2.68	52.5%

AF, relative atrophy factor; AF-FAST, relative type 2 atrophy factor; AF-SLOW, relative type 1 atrophy factor; HF, relative hypertrophy factor, HF-FAST, relative type 2 hypertrophy factor; HF-SLOW, relative type 1 hypertrophy factor.

### Insulin receptor alternative splicing and protein expression

**Fig 2:**
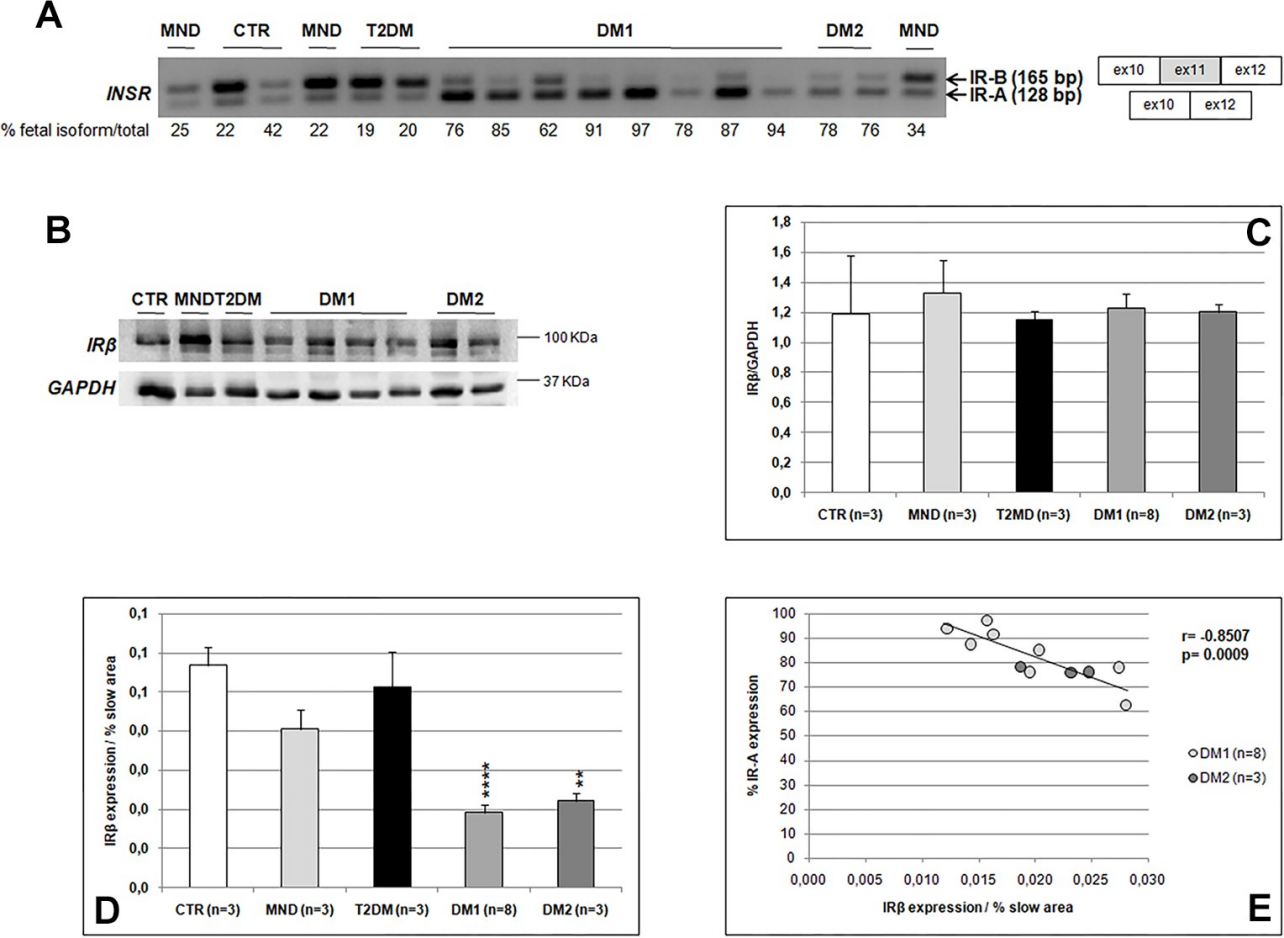
Insulin Receptor alternative splicing and protein expression. **(A)** *INSR* splicing products obtained by RT-PCR amplification of RNA isolated from skeletal muscle biopsies. Bands were quantified and proportions of fetal isoform IR-A (-exon 11) were calculated. **(B)** Representative western blot analysis of the basal expression of IR in skeletal muscle biopsies. **(C)** Histograms represent mean values of IR expression and bars represent standard error of the mean (SEM). Density of the bands have been normalized to GAPDH expression. **(D)** Histograms representing the relative amount of IR expression contributed by type 1 fibers. Density of IR bands has been normalized to the percentage of type 1 fibers area. Bars represent standard error of the mean (SEM). Differences between groups have been evaluated by Student *t*-test. **p<0.01; ****p<0.0001. It is of note that in the graphic only the statistical differences between DM1 or DM2 and CTR group are shown. **(E)** Pearson’s correlation between the percentage of fetal IR-A expression and the relative amount of IR expression contributed by type 1 fibers in DM patients.

To better understand the role of the insulin receptor in DM insulin resistance, its expression was analysed in all skeletal muscle samples used in this study. *INSR* alternative splicing was determined by RT-PCR analysis in DM and non-DM skeletal muscle (Fig 2A). IR-B was the predominant isoform expressed in normal (67.8±9.8%), MND (72.8±3.7%) and T2DM (80.1±0.6%) skeletal muscle. On the contrary, the fetal IR-A isoform consistently predominated in both DM1 (84.0±4.0%) and DM2 (77.0±1.0%).

To study the IR protein expression in DM skeletal muscle a western blot analysis using an antibody against the IRβ subunit was performed. The results showed similar protein expression levels in all samples examined (Fig 2B and 2C), however, this analysis was performed on homogenized muscles that can hide fiber-specific differences in the insulin receptor expression. Thus, serial sections immunostained with IR or MHCslow myosin were compared in order to evaluate possible fiber specific differences in IR expression (Fig 1A–1J). Our results indicate that, in all skeletal muscle examined, IR was expressed in every MHCslow positive fiber (type 1 fibers), while no positive reaction was observed in MHCfast positive fibers (type 2 fibers). Moreover, all type 1 fibers exhibited similar IR immunostaining in both normal and pathological conditions. Thus, in order to evaluate IR protein expression in relation to type 1 fiber content, IR western blot results were normalized on the percentage of type 1 fiber area (Table 2). This evaluation showed that DM1 and DM2 muscles exhibited an evident lower IR expression than CTR (CTR *vs* DM1 p = 0.00005, CTR *vs* DM2 p = 0.002), MND (NMD *vs* DM1 p = 0.001, NMD vs DM2 p = 0.02) and T2DM (T2DM vs DM1 p = 0.0004, T2DM vs DM2 p = 0.02) (Fig 2D). On the contrary, no differences were found between CTR, MND and T2DM muscles. Interestingly, in DM samples a significant negative correlation was found between the relative amount of type 1 fiber-specific IR expression and the percentage of the IR-A fetal isoform RNA expression (Fig 2E). This correlations were statistically significant also when considering all the samples examined (Pearson r = -0.8948, p = 0.0001).

### Validation of the method used to study the activation of insulin pathway in skeletal muscle

To study the activation of insulin pathway in DM skeletal muscle, skeletal muscle samples were treated with insulin using a previously described well-etablished experimental protocol with some modifications [19–21]. To test if the method used in this work leaded to the insulin dependent pathway activation, we analysed the insulin-dependent phosphorylation of IRS1, AKT/PKB, ERK1/2 and p70S6K in 4 *biceps brachii* biopsies obtained from healthy subjects. Among them, 2 patients underwent overnight fasting (CTR-NotFed), while the other 2 had breakfast before muscle sample collection (CTR-Fed). As expected, in CTR-NotFed subjects insulin induced the activation of the pathway indicated by protein phosphorylation, while no insulin stimulation was observed in CTR-Fed subjects (S1 Fig). These results allowed us to confirm that this protocol was useful to analyse the response to insulin action directly on skeletal muscle samples.

### Response to insulin action in DM skeletal muscle biopsies

**Fig 3:**
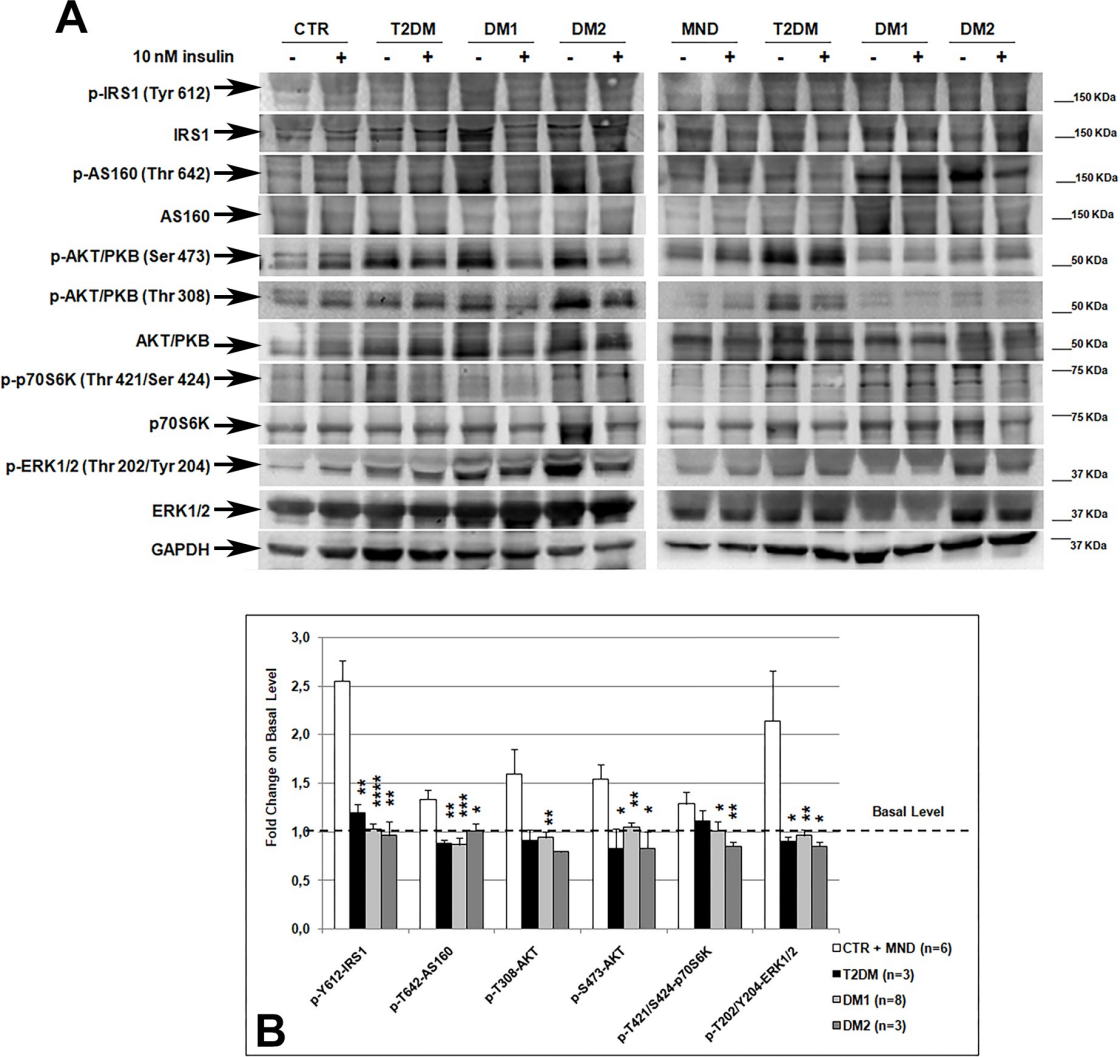
Insulin signalling activation in skeletal muscle biopsies. **(A)** Representative western blot analysis of the expression and phosphorylation of proteins involved in the insulin pathway. Skeletal muscle samples were incubated in absence (-) or presence (+) of 10 nM insulin for 20 minutes. Due to the high number of samples analysed, the figure panel is composed by images of two different gels. Details on how western blot experiments were performed are reported in Materials and Methods. **(B)** Fold change on basal level of the quantification of IRS1, AS160, AKT/PKB, p70S6K and ERK1/2 activation. Histograms represent mean values and bars represent standard error of the mean (SEM). The number of samples analysed in each group is reported in graphic legend. Differences between groups have been evaluated by Student *t*-test. *p<0.05; **p<0.01; ****p<0.0001.

To evaluate the action of insulin stimulation in DM skeletal muscle, the phosphorylation state of key proteins of the two best characterized IRS-1-Akt/PKB and Ras-ERK insulin signalling pathways was analysed by western blot. No differences were found in the activation of the insulin pathway between CTR and MND muscles (Fig 3A). Indeed, western blot analysis showed that in CTR and MND insulin induced a similar increase in the phosphorylation state of IRS1, AS160, AKT/PKB, p70S6K and ERK1/2. Thus, the results of protein activation were pooled in order to increase the number of control patients for statistical analysis (Fig 3). In DM1 and DM2 muscles, insulin induced a statistically significant lower activation of these proteins as compared to CTR+MND (Fig 3). Similar results were observed in T2DM patients (Fig 3).

A positive correlation between type 1 fiber-specific IR expression and activation of some proteins of the insulin pathway was found (IRS1 Pearson r = 0.538, p = 0.02; S473-AKT Pearson r = 0.624, p = 0.007; p70S6K Pearson r = 0.482, p = 0.04; ERK1/2 Pearson r = 0.690, p = 0.001).

### Insulin effect on the regulation of DM skeletal muscle mass

It is known that insulin plays a critical role in the regulation of skeletal muscle mass regulating protein metabolism through two main downstream effectors: mTOR and FoxO. Indeed, insulin induces the phosphorylation and activation of mTOR promoting protein synthesis, while insulin dependent phosphorylation of FoxO1 leads to a decrease in atrogenes expression, such as MuRF1 and Atrogin-1/MAFbx, thus inhibiting ubiquitin-dependent protein degradation [29–32].

**Fig 4:**
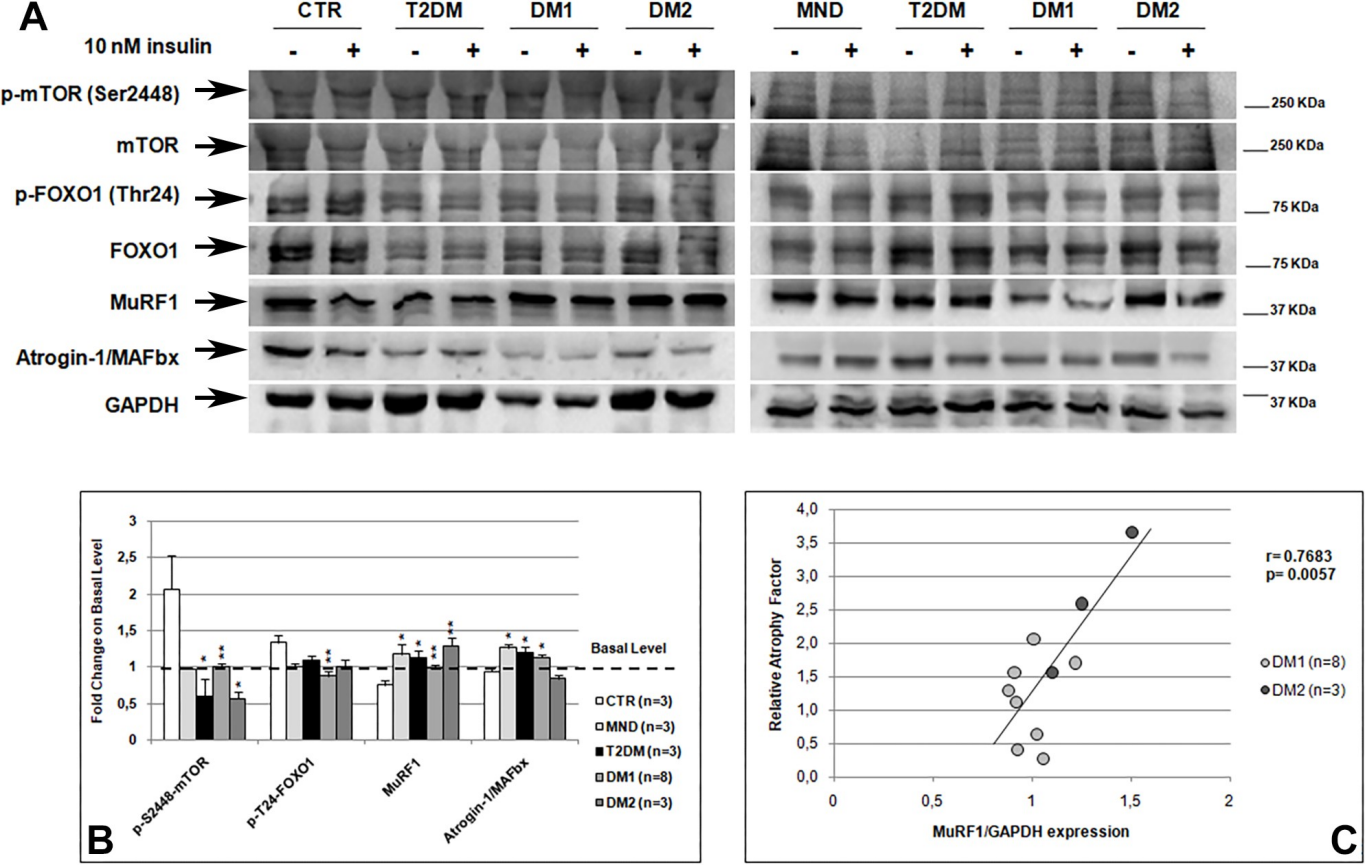
Insulin dependent regulation of skeletal muscle mass. **(A)** Representative western blot analysis of the expression and phosphorylation of proteins involved in insulin dependent regulation of protein metabolism. Skeletal muscle samples were incubated in absence (-) or presence (+) of 10 nM insulin for 20 minutes. Due to the high number of samples analysed, the figure panel is composed by images of two different gels. Details on how western blot experiments were performed are reported in Materials and Methods. **(B)** Fold change on basal level of the quantification of mTOR and FoxO1 activation and of MuRF1 and Atrogin-1/MAFbx expression. Histograms represent mean values and bars represent standard error of the mean (SEM). The number of samples analysed in each group (n) is reported in the graphic legend. Density of MuRF1 and Atrogin-1/MAFbx bands have been normalized to GAPDH expression. Differences between groups have been evaluated by Student *t*-test. *p<0.05; **p<0.01. **(C)** Pearson’s correlation between the relative atrophy factors and MuRF1 expression in DM patients.

As expected, western blot analysis showed that in CTR muscle insulin induced an increase in mTOR and FoxO1 phosphorylation and a decrease in MuRF1 and Atrogin-1/MAFbx expression (Fig 4A and 4B). Interestingly, contrary to what observed for IRS-1-Akt/PKB and Ras-ERK insulin signalling pathways activation, in MND muscle insulin did not lead to an increase in mTOR and FoxO1 phosphorylation, thus a significantly increase in MuRF1 and Atrogin-1/MAFbx expression is observed. Also in DM1 and DM2 skeletal muscle, insulin did not induce an increase in mTOR and FoxO1 phosphorylation, which levels resulted statistically significant lower than those observed in CTR muscle. Therefore, in DM skeletal muscle the lower inactivation of FoxO1 did not induce a decrease in MuRF1 and Atrogin-1/MAFbx expression, which resulted significantly higher than in CTR except for Atrogin-1/MAFbx in DM2 muscle. Similar results were obtained in T2DM skeletal muscle. To verify if alterations in mTOR and FoxO1 phosphorylation observed in our DM and T2DM samples were related to insulin insensitivity, we analysed the correlation between their phosphorylation levels with those of the proteins of IRS-1-Akt/PKB and Ras-ERK pathways. Both mTOR and FoxO1 phosphorylation levels showed a positive significant correlation with the activation of the insulin pathway (mTOR: IRS1 Pearson r = 0.7146, p = 0.001, T308-AKT/PKB Pearson r = 0.5608, p = 0.01, S473-AKT/PKB Pearson r = 0.7989, p = 0.0001, p70S6K Pearson r = 0.6324, p = 0.005, ERK1/2 Pearson r = 0.7923, p = 0.0001, AS160 Pearson r = 0.5123, p = 0.02; FoxO1: IRS1 Pearson r = 0.5099, p = 0.03, T308-AKT/PKB Pearson r = 0.4439, p = 0.05, p70S6K Pearson r = 0.7196, p = 0.0005, ERK1/2 Pearson r = 0.590, p = 0.007). Moreover, a positive correlation between type 1 fiber-specific IR expression and mTOR and FoxO1 phosphorylation was found (mTOR Pearson r = 0.489, p = 0.04; FoxO1 Pearson r = 0.549, p = 0.01).

In order to verify if the altered mTOR and FoxO1 pathways could contribute to skeletal muscle atrophy in DM and T2DM patients i.e. those patients who showed insulin resistance, we analysed the correlation of mTOR, FoxO1, MuRF1 and Atrogin-1/MAFbx expressions with atrophy factors. The results of this analysis showed that, when considering DM and T2DM patients or DM patients alone, a negative correlation was present between mTOR activation and the relative atrophy factor, even if not statistically significant (DM+T2DM: Pearson r = -0.47, p = 0.06; DM: Pearson r = -0.49, p = 0.08). Moreover, MuRF1 expression significantly correlates with the relative atrophy factor and with the type 2 fiber atrophy factor (AF-FAST) when considering T2DM and DM patients or DM patients alone (DM+T2DM: AF Pearson r = 0.59, p = 0.01, AF-FAST Pearson r = 0.48, p = 0.05; DM: AF Pearson r = 0.82, p = 0.0005, AF-FAST: Pearson r = 0.71, p = 0.007) (Fig 4C). No correlation was found between Atrogin-1/MAFbx and atrophy factors when considering DM and T2DM patients. However, when only DM1 and T2DM patients were taken into consideration a positive correlation with the relative atrophy factor was observed (Pearson r = 0.63, p = 0.02).

## Discussion

Myotonic dystrophies are multisystemic diseases characterized by the presence of metabolic dysfunctions such as insulin resistance, hyperinsulinemia, increased fat mass and a fourfold higher increase of developing type 2 diabetes (T2DM). These metabolic dysfunctions can significantly worsen some aspect of the disease, especially at skeletal muscle, brain and heart level, thus worsening the quality of life of these patients. In literature, the studies aimed to define DM insulin resistance driving mechanisms provided evidences that in addition to aberrant splicing of *INSR* gene, defects in post-receptor insulin signalling might contribute to this feature of the disease [10–11,13,29–35]. Moreover, the expression of the insulin receptor in DM skeletal muscles is still controversial, since both normal and diminished RNA and protein levels have been reported [10,34,36–37]. In our work, for the first time we have elucidated the role of IR expression in DM insulin resistance and we have investigated the effects of acute insulin stimulation on the pathways that regulate skeletal muscle mass in DM1 and DM2 patients. Since cultured cells fail to replicate all properties of adult muscle, the ability to analyse insulin signalling activation directly in adult DM muscles would provide more significant results into the knowledge of mechanisms driving insulin resistance and skeletal muscle fiber atrophy.

In order to better elucidate the role of insulin receptor in DM insulin resistance, we have analysed its expression in muscle samples at RNA and protein levels. As expected, our splicing analysis showed that in both DM1 and DM2 muscle biopsies the fetal IR-A isoform was more expressed than the IR-B adult one in all patients, while no alteration of *INSR* alternative splicing was observed in non-DM patients. Similarly to what reported in previous studies [10,34,36–37], when we analysed IR protein expression in the whole homogenized muscles, no differences between DM and control muscles were observed. However, analysis performed on homogenized muscles can hide fiber-specific differences in the insulin receptor expression. Indeed, in line with previous data [38–39], our immunolocalization study of IR showed that the receptor is expressed mainly in type 1 fibers in all samples analysed, since type 1 fibers play an important role for maintaining glucose homeostasis in response to insulin [38,40]. Nevertheless, our analysis of IR protein expression in relation to type 1 fiber content, showed that DM muscles exhibit lower expression levels of insulin receptor as compared to non-DM muscles. These results are similar to those obtained in skeletal muscles of patients affected by metabolic syndrome, where type 1 fibers consistently express lower levels of key elements of insulin action as compared to healthy subjects [38]. This lower expression of IR observed in DM type 1 fibers, could be related to the higher expression of IR-A isoform in DM skeletal muscles since the percentage of the fetal isoform IR-A expression inversely correlates with the relative amount of IR contributed by type 1 fibers. Thus, we can speculate that fetal isoform IR-A translation is lower than that of the adult IR-B. However, we cannot confirm this hypothesis, since no commercial antibodies against the two different IR isoforms are available. The aberrant alternative splicing of the insulin receptor has been associated with insulin resistance in DM patients because of the possible different binding affinity and signaling specificity of the two isoforms for insulin [10]. Our results seem to indicate that in DM skeletal muscle, despite the lower signaling potential of IR-A [41,42], a reduction in IR-A protein synthesis could contribute to DM insulin resistance. Indeed, the positive correlation between IR expression and the activation of the insulin pathway seems indicate that in DM patients the lower activation of insulin signalling could be due to the lower type 1 fiber-specific IR expression.

The results of our analysis of the insulin pathway activation obtained from the insulin stimulation of *ex vivo* DM skeletal muscle biopsies provided evidences for defective insulin signalling in DM1 and DM2 skeletal muscles, confirming what we previously observed in differentiated DM muscle cells [13]. Moreover, our *ex vivo* study allowed us to confirm that in DM skeletal muscle a higher basal level of protein phosphorylation is present as already reported by our group [13]. These results were supported by data obtained in T2DM muscles which also do not show signalling activation after insulin stimulation. Among proteins analysed, particularly interesting are data regarding AS160. Indeed, since the little amount of skeletal muscle biopsies available for this study did not allowed us to perform the evaluation of glucose uptake during insulin stimulation, we have analysed the activation state of AS160, a Rab GTPase-activating protein which is involved in insulin dependent GLUT4 translocation into the plasma membrane, where glucose uptake takes place [43–45]. In physiological conditions, AS160 is phosphorylated on Thr642 in response to insulin, leading to the dissociation of the protein from cytosolic vesicles and to GLUT4 membrane translocation [46]. The absence of insulin dependent activation of AS160 in DM patients might explain the lower increase of glucose uptake observed in DM muscle cells after acute insulin stimulation [13]. These results support the hypothesis that impaired glucose uptake in DM skeletal muscle might be possibly explained by defects in insulin stimulated translocation of GLUT4 storage vesicles and not by a defective synthesis of the transporter. It should be noted that our DM2 samples exhibited a lower pathway activation as compared to DM1, confirming what was previously observed in DM skeletal muscle cells [13]. These results are consistent with the higher risk to develop type 2 diabetes observed in DM2 patients [47]. Taken together these results confirm that DM skeletal muscle exhibits a lower insulin sensitivity, probably due to the high levels of basal protein phosphorylation that leads to a lack of further insulin stimulated phosphorylation [13].

At skeletal muscle level, there is still no mechanistic explanation for muscle weakness and wasting observed in DM patients or for the muscle histopathological features characteristic of this disease. It is known that insulin pathway plays a central role in regulating muscle mass through its ability to modulate protein synthesis, autophagy, ubiquitin-mediated protein degradation and myogenesis [48]. In order to understand if there is a relation between insulin resistance and muscle wasting in DM patients, we used an experimental approach that allowed us to analyse those pathways involved in skeletal muscle mass regulation and histopathological features on the same muscle sample. Binding of insulin to its receptor leads to AKT/PKB activation and mTOR and FoxO phosphorylation. Once phosphorylated, mTOR is activated and allows for the initiation of translation and acceleration of protein synthesis, while FoxO1 phosphorylation leads to the inhibition of MuRF1 and Atrogin-1/MAFbx transcription, two ubiquitin ligases that mediated ubiquitin-dependent protein degradation [49–50]. Our observation of a lower phosphorylation of mTOR and FoxO1 in DM and T2DM patients seems indicate that in skeletal muscle an alteration in protein metabolism is present. This imbalance between the rates of protein synthesis and degradation might be due to the lack of insulin pathway activation observed in these patients. Indeed, our analysis showed that the lower expression of the insulin receptor and the lower insulin dependent AKT/PKB activation observed in DM skeletal muscle are accompanied by a lower phosphorylation of mTOR and FoxO1. Moreover, when considering insulin insensitive skeletal muscles, i.e. DM and T2DM, our results seem indicate that the presence of atrophic fibers in skeletal muscle might be due to an imbalance between the rates of muscle protein synthesis and degradation. Indeed, mTOR activation negatively correlates with atrophy factors, while MuRF1 expression directly correlates with skeletal muscle fiber atrophy. Interestingly, the increase in MuRF1 expression significantly correlates with type 2 fiber atrophy factors and our DM2 patients, which are characterised by a selective type 2 fiber atrophy, showed the higher levels of MuRF1 expression. These results are in line with the observation that FoxO1-related muscle atrophy primarily affects type 2 fibers through the regulation of MuRF1 [51]. Indeed, Atrogin-1/MAFbx correlates with atrophy factors only when considering DM1 and T2DM patients, who show both type 1 and type 2 fiber atrophy. The possible involvement of atrogenes expression in DM skeletal muscle atrophy, is supported by our data obtained in MND skeletal muscle, which also exhibit an increase in MuRF1 and Atrogin-1/MAFbx related to skeletal muscle atrophy [50,52–53]. Our results on the aberrant mTOR/FoxO1 pathways in DM muscles are in line with those observed by other authors that showed an abnormal mTORC1 signalling activation in HSA^LR^ mice and in human DM1 cells [33,54,55], and an increase in proteasome activity in DM2 muscle cells [18,56]. Interestingly, it should be noted that mTOR is also a key modulator of autophagy and several studies have shown that autophagy could be involved in the degenerative loss of muscle tissue in DM skeletal muscle [54,55,57,58].

In conclusion, although a limit of this study is the low number of patients examined, our results added new mechanistic insights into insulin resistance and muscle fiber atrophy in DM patients. We have provided for the first time evidences of a lower expression of the insulin receptor in DM muscle that lead to a lower insulin signalling activation and thus to an alteration of the balance between protein synthesis and degradation. Nowadays there is no treatment or cure for myotonic dystrophies and developing therapies for the prevention and treatment of insulin resistance condition and muscle atrophy process will enhance the quality of life of patients who suffer from this disease. Indeed, metabolic changes contribute to muscle weakness and wasting, cardiovascular diseases and neuropathies, thus, the identification of therapeutic target for insulin resistance treatment in DM patients could contribute to ameliorate the multisystemic spectrum of these diseases.

## Supporting information

S1 Fig Insulin signalling activation in healthy subjects. **(A)** Representative western blot analysis of the expression and phosphorylation of proteins involved in the insulin pathway in healthy subjects that underwent overnight fasting (CTR-NotFed) or not (CTR-Fed). Skeletal muscle samples were incubated in absence (-) or presence (+) of 10 nM insulin for 20 minutes. **(B)** Fold change on basal level of the quantification of IRS1, ERK1/2, AKT/PKB and p70S6K activation. Histograms represent mean values and bars represent standard error of the mean (SEM). The number of samples analysed in each group (n) is reported in graphic legend.
(TIF)
