# Supplementary figures and images for: Aberrant insulin receptor expression is associated with insulin resistance and skeletal muscle atrophy in myotonic dystrophies
Source: PLoS One. 2019 Mar 22;14(3):e0214254. doi: 10.1371/journal.pone.0214254 (PMC6430513; doi:10.1371/journal.pone.0214254)

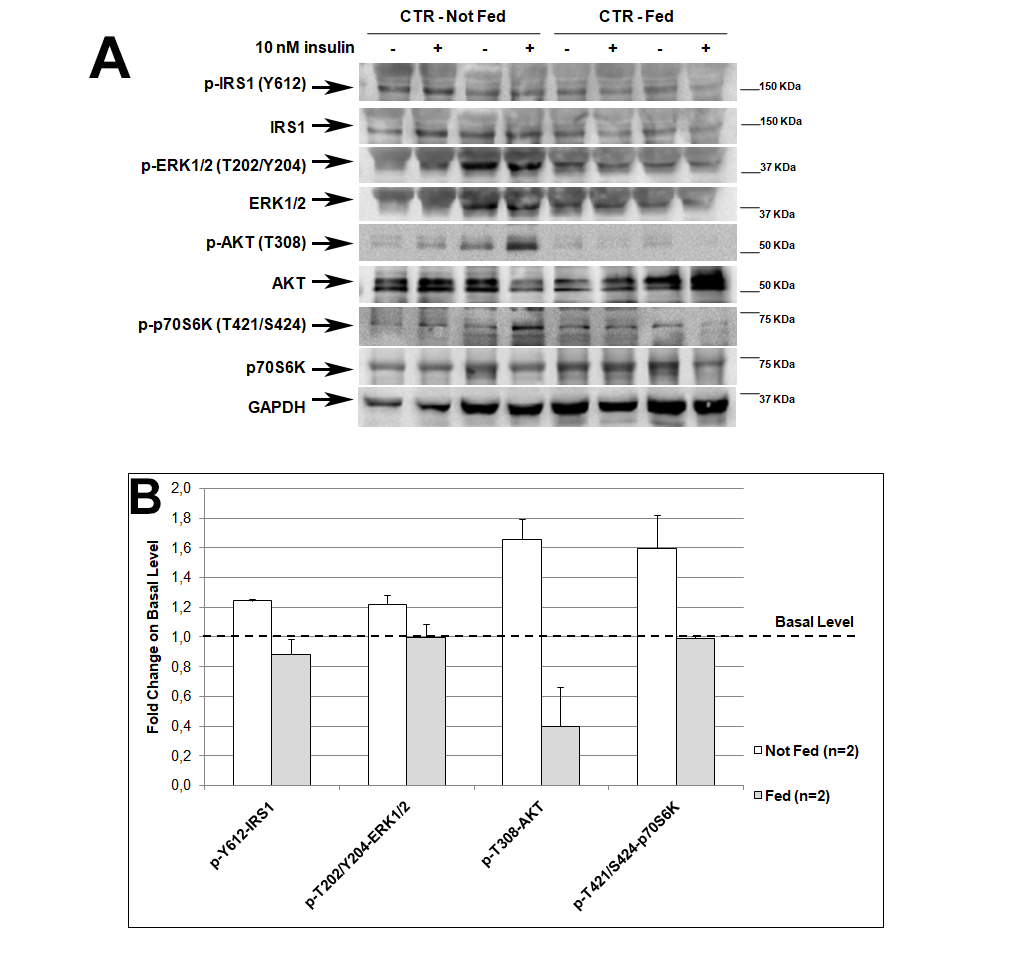

Supplement: S1 Fig — (A) Representative western blot analysis of the expression and phosphorylation of proteins involved in the insulin pathway in healthy subjects that underwent overnight fasting (CTR-NotFed) or not (CTR-Fed). Skeletal muscle samples were incubated in absence (-) or presence (+) of 10 nM insulin for 20 minutes. (B) Fold change on basal level of the quantification of IRS1, ERK1/2, AKT/PKB and p70S6K activation. Histograms represent mean values and bars represent standard error of the mean (SEM). The number of samples analysed in each group (n) is reported in graphic legend. (TIF) [file pone.0214254.s001.tif]
